# Spectral Efficiency Improvement Using Bi-Deep Learning Model for IRS-Assisted MU-MISO Communication System

**DOI:** 10.3390/s23187793

**Published:** 2023-09-11

**Authors:** Md Abdul Aziz, Md Habibur Rahman, Mohammad Abrar Shakil Sejan, Jung-In Baik, Dong-Sun Kim, Hyoung-Kyu Song

**Affiliations:** 1 Department of Information and Communication Engineering, Sejong University, Seoul 05006, Republic of Korea; aziz@sju.ac.kr (M.A.A.); habibur@sju.ac.kr (M.H.R.); sejan@sejong.ac.kr (M.A.S.S.); junginb@sejong.ac.kr (J.-I.B.); 2 Department of Convergence Engineering for Intelligent Drone, Sejong University, Seoul 05006, Republic of Korea; 3 Department of Semiconductor Systems Engineering, Sejong University, Seoul 05006, Republic of Korea; dongsun.kim07@sejong.ac.kr

**Keywords:** IRS, machine learning, Bi-LSTM, spectral efficiency, multi-user multiple input single output

## Abstract

The intelligent reflecting surface (IRS) is a two-dimensional (2D) surface with a programmable structure and is composed of many arrays. The arrays are used to supervise electromagnetic wave propagation by altering the electric and magnetic properties of the 2D surface. IRS can influentially convert wireless channels to very effectively enhance spectral efficiency (SE) and communication performance in wireless systems. However, proper channel information is necessary to realize the IRS anticipated gains. The conventional technique has been taken into consideration in recent attempts to fix this issue, which is straightforward but not ideal. A deep learning model which is called the long short-term memory (Bi-LSTM) model can tackle this issue due to its good learning capability and it plays a vital role in enhancing SE. Bi-LSTM can collect data from both forward and backward directions simultaneously to provide improved prediction accuracy. Because of the tremendous benefits of the Bi-LSTM model, in this paper, an IRS-assisted Bi-LSTM model-based multi-user multiple input single output downlink system is proposed for SE improvement. A Wiener filter is used to determine the optimal phase of each IRS element. In the simulation results, the proposed system is compared with other DL models and methods for the SE performance evaluation. The model exhibits satisfactory SE performance with a different signal-to-noise ratio compared to other schemes in the online phase.

## 1. Introduction

Intelligent reflecting surface (IRS) is a promising technology for future wireless systems in the internet of things (IoT)-based 5th-generation (5G) and 6G communication. In IRS-assisted communication, an expected reflection pattern of the transmitted signal can be obtained by adjusting the phase shift of the IRS elements. IRS-assisted communication is characterized by various benefits such as easy operation, reduced cell edge outages, and a plaintively re-configurable system with passive beamforming (PB), which is made possible via joint optimization of the phase shift [1]. Recently, the application of IRS has dramatically increased in the area of non-line of sight (LOS) communication methods like mobile edge computing, power transfer, unnamed aerial vehicles, enhanced physical layer security, IoT applications, and interference cancellation by optimally varying the reflection coefficient [2]. IRS is used as a bypass element when LOS communication is not available between the base station (BS) and user equipment (UE). IRS can cancel the unwanted signal and can enhance physical layer security by utilizing PB [3]. In addition, by designing a suitable wireless channel, IRS increases data transmission efficiency with less power consumption and boosts information security. Some IRS-assisted work has been conducted to reduce system power consumption and boost information security [4,5,6,7]. Due to the striking advantages and applications of IRS in wireless systems, many types of research have been performed for the analysis of SE [8,9], energy efficiency [10], channel estimation [11], achievable rate [12], and so on.

In recent years, machine learning (ML) has been extensively used in the area of IRS-assisted wireless communication [13,14,15,16,17]. In IRS-assisted communication, channel state information (CSI) acquisition is a big challenge and can be easily overcome by using deep learning (DL)-based models. The performance of wireless communication systems is degraded in several cases as a result of the nonlinearity and imperfection of the system. Due to the non-linearity characteristic of the IRS component, a non-convex problem is created and the system suffers from a channel rank problem [18,19,20,21]. Besides, developing a mathematical model for IRS-based wireless communication is very challenging. The DL model can be used to alleviate the above issues. Furthermore, the DL-based approach does not need to calculate the complex mathematical model and easily optimizes the channel imperfection. DL-based IRS technology is used to extract the internal relationship between the input and output signals and helps to achieve better SE compared to the traditional model-based scheme.

A deep reinforcement learning (DRL) model-based IRS-assisted MU-MISO communication system was proposed in [22]. In [23], the authors proposed an IRS-assisted downlink MISO scenario with unsupervised DRL models (deterministic policy gradient (DDPG) and twin delayed DDPG (TD3)) for SE analysis. A deep neural network (DNN) based IRS-aided PB was proposed in [24] to improve SE. In [25], the authors proposed a SE maximization method using ML in a massive multiple input–multiple output (MIMO) system. The authors of [25] used gradient boosting and NN models to measure SE with different mean average percentage error (MAPE) calculations. A multi-IRS-aided massive MIMO non-orthogonal multiple access (NOMA) network was designed in [26] to measure the SE. The authors of [26] applied an adaptive user pairing scheme to maximize the SE with a DNN model-based joint optimization technique.

DL-based methods are also capable of classifying sequence data using recurrent neural networks (RNNs). For IRS-assisted MIMO communication, deep convolutional and RNNs-based optimal decoding was proposed in [27]. Long short-term memory (LSTM) is a kind of RNN known as an efficient dynamic classifier. It is made up of several memory cells with the capacity to store and update data over extensive time steps. LSTM uses gates to regulate information flow and prevents the gradient vanishing or exploding problem. Long-term dependencies and sequential patterns in the data can be captured using LSTM [28,29,30]. Many works have been created using the LSTM model in wireless communication [31,32,33,34]. However, IRS-assisted wireless communication has been discussed in only a limited number of studies with regard to LSTM-based SE measurement. The authors of [33] proposed an LSTM-based energy-efficient IRS communication system for establishing harvesting to improve the SE. In [35], the authors worked on IRS-assisted LSTM-based SE evaluation by monitoring wireless terminal movement. Using the estimated channel as time-series data, the authors suggested an evaluation method that considered wireless terminal movement.

**Figure 1:**
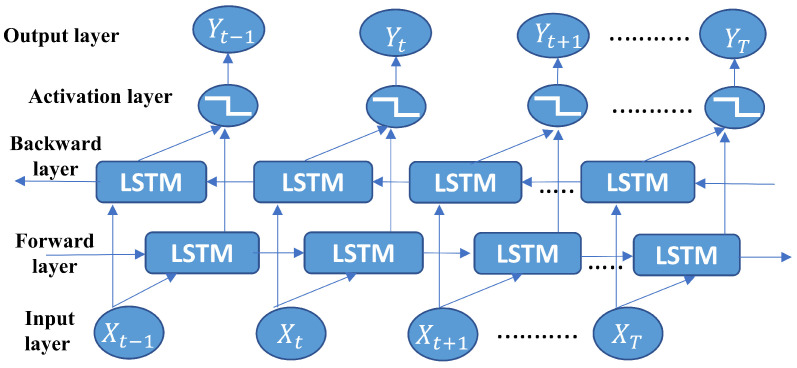
Input information receiving architecture of Bi-LSTM model.

Bi-directional long short-term memory (Bi-LSTM) consists of two LSTMs: one taking input data in the forward direction and another in the backward direction. Bi-LSTM is efficient in sequence classification, allowing the data to move in both forward and backward directions. For the Bi-LSTM model, hidden layers can receive feature information from both the past and future simultaneously. Therefore, Bi-LSTM provides extra training capability, better prediction accuracy, and information availability [36]. For this reason, our proposed Bi-LSTM model outperforms other deep learning models. The basic structure of a Bi-LSTM model is illustrated in Figure 1. A Bi-LSTM hidden layer consists of two layers such that one set of LSTM layers connected in the forward direction is called the forward layer, and another set of LSTM layers connected in the backward direction is called the backward layer. In summary, Bi-LSTM can be an important candidate for IRS-based SE improvement as it can extract more features from data in two directions. The aforementioned application and benefits of Bi-LSTM in the wireless communication system are important specially for IRS-based communication [16].

Phase shift optimization also plays an important role for IRS-based communication. There are many works that have been created to optimize the IRS phase shift by applying different optimization algorithms. In [37], the authors proposed the joint optimization of BF vectors, power, and IRS phase shift using an alternating algorithm based on a partial exhaustive search. In [38], the authors proposed the joint optimization of the IRS phase shift matrix, BS vectors, and NOMA user ordering. They applied a difference-of-convex (DC) algorithm to optimize the IRS phase shift. In [39], a joint deployment of phase shift, power allocation control, and dynamic decoding order was proposed by the authors. In addition, the authors applied a decaying double deep Q-network $\left(D^{3}QN\right)$ algorithm. In this paper, we have configured a Wiener filter optimization algorithm to optimize the IRS phase shift and also proposed a Bi-LSTM model-based SE analysis scheme in the IRS-assisted MU-MISO system.

The major contributions of the proposed system can be summed up as follows:

- A highly efficient DL-based SE analysis in the IRS-assisted MU-MISO downlink system is proposed, where a Wiener filter is employed to optimize the phase shift vector in the IRS elements during communication establishment.
- The received simulated channel data are fed to the DL model, which is called the Bi-LSTM model during training. Due to the forward and backward training ability of Bi-LSTM hidden layers, it reduces the losses and improves the SE prediction in the online phase. To optimize the loss during training, the Adam optimizer is deployed.
- To evaluate the effectiveness of the proposed model in terms of SE analysis and different signal-to-noise ratios (SNRs), comparative simulation outcomes are performed with alternative optimization, DL-based BF, DDPG, and TD3 models, respectively. The simulation results indicate that the proposed model outperforms the other methods very effectively.

The rest of the paper is organized as follows. Section 2 describes the system modeling. Section 3 describes the proposed model in detail. Section 4 shows the simulation outcomes and, finally, the conclusions are represented in Section 5.

## 2. System Model

**Figure 2:**
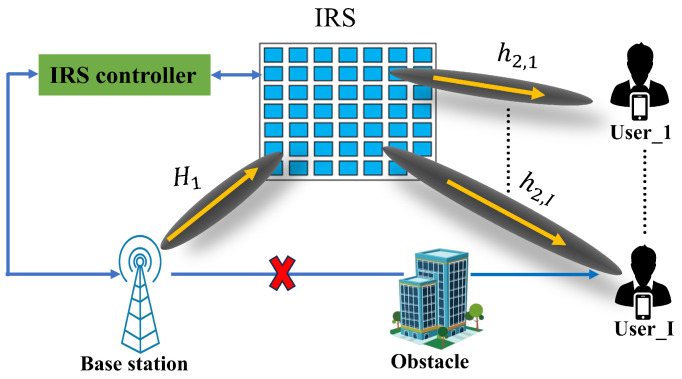
IRS-assisted downlink MU-MISO communication system.

The proposed IRS-assisted downlink MU-MISO communication system is shown in Figure 2. It is considered that the direct path from BS to UEs is blocked by obstacles. Thus, in the paper, we assumed the cascade channels from BS to IRS and IRS to UEs are used to transmit the BS information to the UEs. Additionally, for the channel state information (CSI) exchange, the IRS controller uses separate wireless lines to connect with the BS and UEs.

### 2.1. Signal Transmission Model

In this paper, the IRS consists of *N* passive reflecting elements, and these elements are arranged as a uniform planar array (UPA). BS contains with *M* uniform linear array (ULA) antennas, and UEs comprise a single antenna with *I* users. Therefore, the received signal at UE, i=1,2,....,I can be written as follows:

(1) $$y_{i}=h_{2,i}^{H}\Phi H_{1}x+n_{i},$$

where $h_{2,i}\in C^{N\times 1}$ represents the IRS to the *I*th UE channel vector and $H_{1}\in C^{N\times M}$ denotes the BS to IRS channel matrix. $\Phi =diag(e^{j\varpi_{1}},e^{j\varpi_{2}},\ldots ,e^{j\varpi_{N}})$ specifies the IRS phase shift matrix, and $\varpi_{n}\in \left[0,2\pi \right]$ identifies the phase shift variable of the *n* reflecting elements. Similarly, $H_{2}$ is the channel matrix between IRS and UEs, which are formulated as $H_{2}$=${\left[h_{2,1}h_{2,2},\ldots ,h_{2,I}\right]}^{H}\in$$C^{I\times N}$. The transmitted signal x is represented as $x=\sum_{i=1}^{I}v_{i}s_{i}$, where $s_{i}∼\mathrm{CN}\left(0,1\right)$ is the *I*th user data symbol, $V=\left[v_{1},v_{2},\ldots ,v_{I}\right]\in C^{M\times I}$ represents the precoding matrix, $n_{i}∼\mathrm{CN}\left(0,\sigma^{2}\right)$ is additive white Gaussian noise (AWGN) and $\sigma^{2}$ is noise variance.

### 2.2. Channel Model

It is assumed that the direct path of BS to UEs is disregarded due to the huge propagation loss. In this paper, we consider the mmWave propagation characteristic and due to this characteristic, a three-dimensional (3D) Saleh–Valenzuela channel model is performed with *L* scatters. Thereafter, the channel $H_{1}$ between BS and IRS is defined as follows:

(2) $$H_{1}=\sqrt{\frac{M\;N}{L}}\sum_{l=0}^{L-1}\beta_{l}a_{u}\left(\phi_{u,l},\theta_{u,l}\right)\;a_{b}^{H}\left(\phi_{b,l}\right),$$

where $\sqrt{\frac{M\;N}{L}}$ represents the normalization factor, $\beta_{l}∼\mathrm{CN}\left(0,1\right)$ is the complex gain of the *l*th scatter. $\;a_{b}^{H}\left(\phi_{b,l}\right)$ and $a_{u}\left(\phi_{u,l},\theta_{u,l}\right)$ denotes the array response vector of BS and IRS. In addition, $\left(\phi_{u,l}\right)$, $\left(\theta_{u,l}\right)$ and $\left(\phi_{b,l}\right)$ represent the azimuth angles of arrival (AOA) and departure (AOD) and the zenith angles of departure (ZOD), respectively. According to [40], the following model describes the array response vector of the UPA with *N* reflecting elements as follows:

(3) $$\begin{array}{c} a_{u}\left(\phi ,\theta \right)=\frac{1}{\sqrt{N}}\left[1,\;\ldots ,\;e^{j\frac{2\pi }{\lambda }d\left(\beta \sin\phi \sin\theta +\gamma \cos\theta \right)},\right.\\ {\left.\ldots ,\;e^{j\frac{2\pi }{\lambda }\left(\left(N_{x}-1\right)d\sin\phi \sin\theta +\left(N_{y}-1\right)\cos\theta \right)}\right]}^{T}, \end{array}$$

where β and γ denote the reflecting elements in the horizontal and vertical directions, $N=N_{x}N_{y}$, $0\leq \beta \leq N_{x}-1$ and $0\leq \gamma \leq N_{y}-1$. The distance between reflective components is *d*, and λ is the wavelength of the signal. The expression for the array response vector of the ULA connected to the BS can be presented as follows:

(4) $$a_{b}\left(\phi \right)=\frac{1}{\sqrt{M}}{\left[1,e^{j\frac{2\pi }{\lambda }d\sin\phi },\ldots ,e^{j\frac{2\pi }{\lambda }\left(M-1\right)d\sin\phi }\right]}^{T}.$$

The channel $h_{2,i}$ between the IRS and UEs can be mathematically formulated as follows:

(5) $$h_{2,i}^{H}=\sqrt{\frac{N}{L_{2}}}\sum_{l^{\prime }=0}^{L_{2}-1}\beta_{l^{\prime }}^{2}a_{b}^{H}\left(\phi_{b,l^{\prime }}^{2},\theta_{b,l^{\prime }}^{2}\right).$$

where $\sqrt{\frac{N}{L_{2}}}$ represents the normalization factor, $\beta_{l^{\prime }}^{2}∼\mathrm{CN}\left(0,1\right)$ is the complex gain of *l*th scatter, and $L_{2}$ identifies the number of paths between IRS and UEs. Finally, the effective cascade channel $H_{eff}$ from BS to IRS and IRS to UEs can be written as follows:

(6) $$H_{eff}=H_{2}\phi H_{1}+n_{i}.$$

However, the total downlink SE *X* of the proposed system can be represented as follows:

(7) $$X=\sum_{i=1}^{I}\log\left(1+\frac{{\left|H_{eff}v_{i}\right|}^{2}}{\sum_{l\neq i}^{}{\left|H_{eff}v_{l}\right|}^{2}+\sigma^{2}}\right).$$

### 2.3. Computation of Optimal Phase Shift Using Wiener Filter

A well-known filter used in signal processing is the Wiener filter, which estimates the best signal or response from a noisy or distorted input [41]. The ideal phase shift vector for a wireless communication system with reflecting components is calculated using the Wiener filter. Reflecting components can be used in wireless communication systems to change the propagation environment and enhance the signal quality at the receiver. In order to enhance the quality of the received signal, the ideal phase shift vector specifies the phase shifts that are applied to the signals that are reflected off the reflecting elements. The ideal phase shift vectors for each sample, reflecting element, and receiver antenna are contained in the calculated W matrix of shape (*S* = number of samples, *N*, *I*). In order to improve the overall received signal quality in the wireless communication system, these vectors can be utilized to set the phase shifts applied to the reflected signals from the reflecting devices. Algorithm 1 shows the utilized Wiener filter in this study.
**Algorithm 1** Optimal phase shift using Wiener filter**Input:***S*: the number of samples*N*: the number of reflecting elements*I*: the number of receiver antennasR: array of shape (*S*, *I*, *I*) representing the auto-correlation of the received signal$H_{eff}$: array of shape (*S*, *N*, *I*) representing the channel gain matrix**Output:**W: array of shape (*S*, *N*, *I*) containing the optimal phase shift vectors**Algorithm:**1: Initialize an array W of shape (*S*, *N*, *I*) with complex zeros.2: For *i* = 0 to *S* − 1:Compute the optimal phase shift vector for sample *i*:(i). Compute the inverse of the matrix R[i].(ii). Compute the complex conjugate transpose of the matrix $H_{eff}\left[i\right]$.(iii). Multiply the inverse of R[i] with the complex conjugate transpose of $H_{eff}\left[i\right]$.(iv). Assign the resulting matrix to W[i].3: Return W.

## 3. Deep Learning Model

### 3.1. Data Generation

To train the proposed model, firstly, we randomly generate the channel matrix data. The channel matrices, which have different sizes for the receiving antenna and the transmitting antenna, respectively, describe the complex channel coefficients between the transmitter and reception antennas. A total of 10,000 samples are taken for training data generation. To generate the SE analysis data, the considering SNR range is from 0 dB to 30 dB with 5 dB intervals. The SE is determined for each SNR value in the preset SNR range, and input–output pairs are gathered for the BiLSTM model. The real and imaginary components of the channel matrix and the real and imaginary components of the phase shift vector are included in the input data. The matching SE numbers are contained in the output data. However, the total input dataset size of 70,000 is generated. According to the system environment, the input data shape is (70,000, 8, 18) and the output data shape is 70,000. The generated data are divided into three subsets: training, testing, and validation. After the data have been properly generated, they are saved to train the model.

### 3.2. Proposed Model Structure

**Figure 3:**
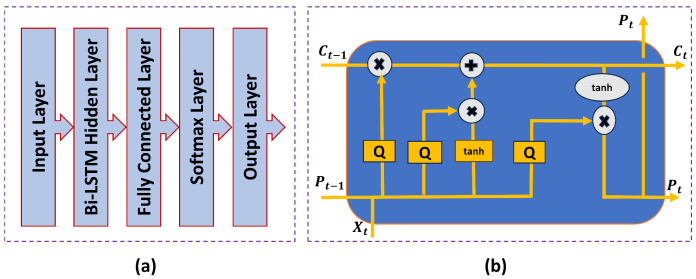
The proposed model structure: (**a**) the structure of different layer workflows of the model; (**b**) the internal cell of LSTM.

Figure 3a,b, respectively, show the structure of different-layer workflows of the proposed model and internal cell. Figure 3b illustrates that a Bi-LSTM model is designed with five layers. (1) an input sequence data layer; (2) a Bi-LSTM hidden layer; (3) a fully connected layer; (4) a softmax layer; and (5) an output layer. Furthermore, the Bi-LSTM hidden layer contains double LSTM layers (forward and backward layers). Each LSTM hidden layer contains 400 hidden units for implementation. The recurrent weight *T*, bias *b*, and input weight *Q* are the learnable weights in the hidden layer. Sequencing and time-series data are employed in the second layer, which is fully connected for classification. The bias vector *b* is added to the input by a fully connected layer after the input has been multiplied by a weight matrix *Q*. During each time step, the fully connected layer performs independently because the input to the hidden layer is a sequence. As a result, it determines each UE’s individual complex modulated signal components. The outputs for the terminal layer are created using the softmax layer. Using the classification layer, the output is converted to a vector probability, and the fully connected layer’s output size is configured to be equal to the number of classes. The training layer then receives feedback from the difference between these two layers.

In Figure 3b, there are three gates included in each LSTM cell [42]. The gates are an input gate, a forget gate, and an output gate. Additionally, LSTM has two states. One is cell state $C_{t-1}$ and another is hidden state $P_{t-1}$. The cell state serves as a memory to store extrapolated data from earlier inputs. It makes use of the hidden state to compute the output. $X_{t}$ is the current input and *t* represents the time instant. The Bi-LSTM cell can add or remove data at each time step from the cell state which is upgraded by the operation of the gate. The level of cell state that needs to be updated and reset is managed, respectively, by the forget gate and input gate. A brief summary of the internal operation of a Bi-LSTM model is shown in Algorithm 2. Also, Table 1 shows the notation of each parameter used in Algorithm 2.
**Algorithm 2** Internal performance of Bi-LSTM model1: **Start**2: The forget gate operation [43]: $fg_{t}$ = $f_{\psi }\left(Q_{fg}X_{t}+T_{fg}P_{t-1}+b_{fg}\right)$3: The input gate operation: $ig_{t}$ = $f_{\psi }\left(Q_{ig}X_{t}+T_{ig}P_{t-1}+b_{ig}\right)$4: The candidate gate operation [43]: $cn_{t}$ = $f_{tanh}\left(Q_{cn}X_{t}+T_{cn}P_{t-1}+b_{cn}\right)$5: The output gate operation: $og_{t}$ = $f_{\psi }\left(Q_{og}X_{t}+T_{og}P_{t-1}+b_{og}\right)$6: Updating the prior cell state, $C_{t-1}$ to the present cell state: $ud_{t}$ = $\left(C_{t-1}⨀fg_{t}\right)+\left(ig_{t}⨀cn_{t}\right)$7: At time step *t*, the hidden state: $P_{t}=og_{t}⨀f_{tanh}\left(ud_{t}\right)$8: The Bi-LSTM layer’s output hidden state: $HI_{t}=f\left(Q_{HI\vec{T}}{\vec{T}}_{t}+Q_{HI\overset{\leftarrow }{T}}{\overset{\leftarrow }{T}}_{t}+b_{z}\right)$9: **Stop**

### 3.3. Training and Testing Procedure

**Figure 4:**
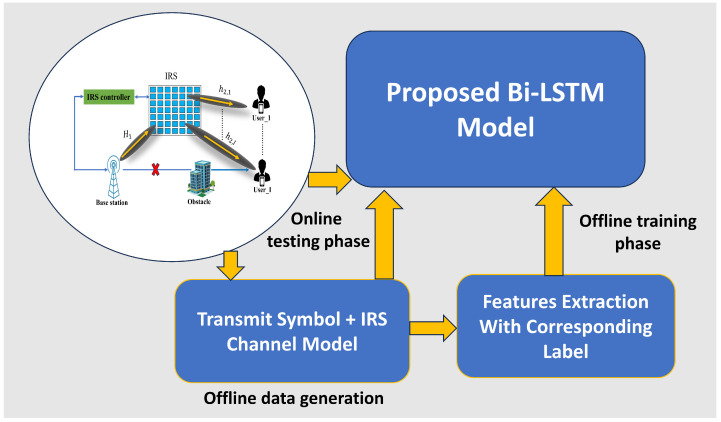
The architecture of proposed Bi-LSTM model with offline training and online testing phase.

**Figure 5:**
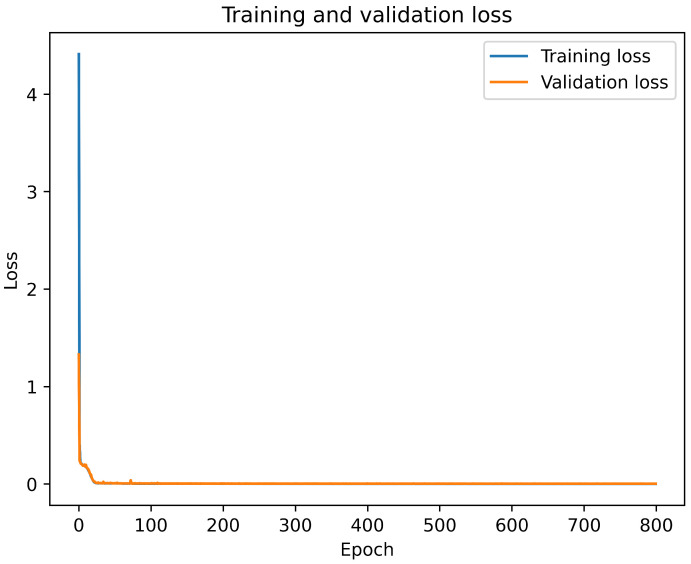
The comparative training and validation loss of the proposed model.

The offline training and online testing process are shown in Figure 4. The data samples are collected during the data generation process and split into three portions: training, testing, and validation. The ratio training portion is 60%, the testing data portion is 20%, and the validation data portion is 20% of the total samples. Then, the training and validation data are loaded as input to the Bi-LSTM model. Algorithm 3 shows the whole training process of the proposed model. The comparative training and validation losses are shown in Figure 5 with an epoch of 800, a minibatch size of 200, and a 0.001 learning rate, respectively. After the offline training is complete, the online testing is performed using a testing dataset. The outstanding simulation result is shown below in the simulation result section.

According to Figure 5, the initial training loss is very high compared to the validation loss. But when the model starts learning, the training loss is decreased along with the validation loss. Within a very short time, the training and validation losses are almost the same, and both are very low. Thus, it is shown that the model can learn in a short time.
**Algorithm 3** Bi-LSTM model training with simulation data1: Training and validation data samples are loaded.2: Initialize the parameter of the model, like minibatch size, learning rate, maximum epoch, and Bi-LSTM layer.3: Actively train the model network4: Calculate the accuracy error of the model.5: Compute the corrective parameter using the Adam optimization algorithm and adjust the parameters while looking for the optimal solution.6: Outcomes: Trained model.7: Saved the model.

## 4. Simulation Results

The performance of the proposed model is presented in this section. In the previous section, data generation, model training, and testing were discussed. The proposed system performs an SE analysis process utilizing the effective parameters shown in Table 2.

**Figure 6:**
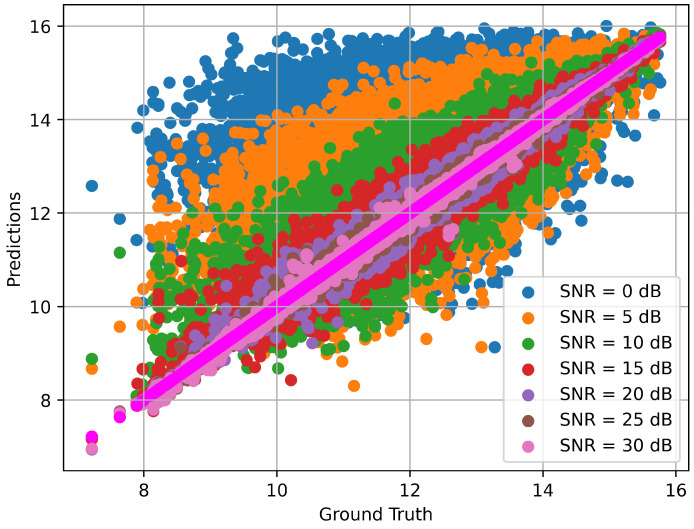
Model prediction accuracy with ground truth for different SNRs.

**Figure 7:**
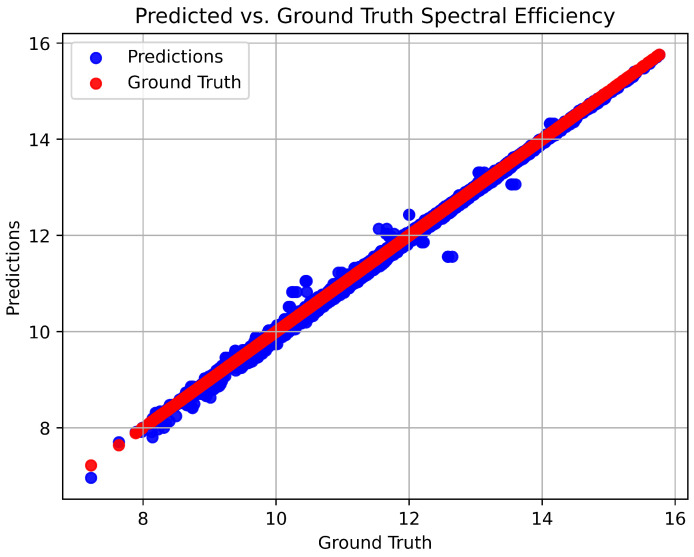
Predicted versus ground truth spectral efficiency.

In this system, the resultant SE is measured with respect to different SNR values. During the generation of the training datasets, the SNR value of 0 to 30 dB is set. During the testing period, the trained model can predict the testing data depending on its previous knowledge. The SE prediction performance is taken with the different SNR values. The matching of the prediction and the channel ground truth with the performance of SNR ranges (0–30) of the proposed model is illustrated as shown in Figure 6. From Figure 6, it is depicted that the prediction capability of the proposed model increases with the SNR value. For SNR 0 dB, the prediction accuracy is low, but the prediction capacity of the model increases with 30 dB SNR. The comparative SE prediction of the proposed model predicted values versus ground truth values is shown in Figure 7. Notably, the predicted values of the proposed model are almost matched with the tested ground truth values, which demonstrates the model’s robustness in terms of prediction performance.

**Figure 8:**
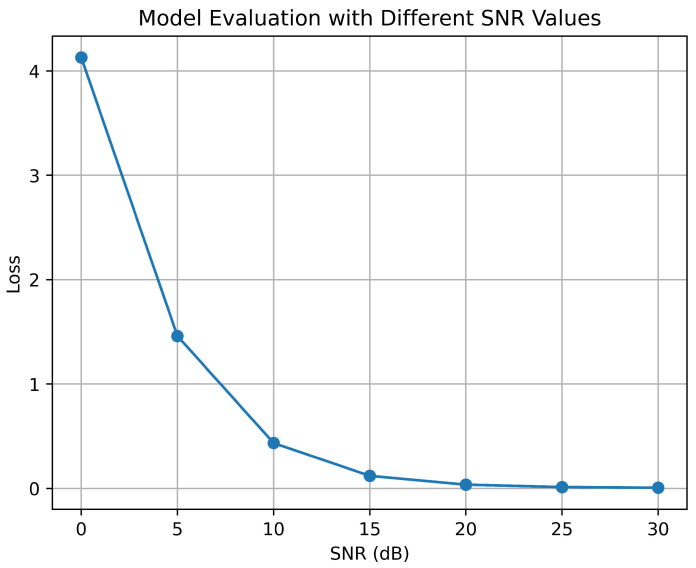
Model evaluation performed by calculating loss with different SNR values.

A DNN model learns from training data how to convert a set of inputs into a set of outputs. In this experiment, the optimization approach of adaptive moment estimation is used to train the model. The goal of this optimization is to reduce the loss during training of the model. To test the minimization of loss, the proposed model uses different SNR values to the ranges of (0–30) dB. The loss versus different SNR curves is shown in Figure 8. The graph shows that the loss of prediction is decreased with respect to the increase in SNR values. For 0 dB SNR, the loss is more than 4; for 20 dB SNR, it is nearly 0; and for 30 dB SNR, it is almost 0.

**Figure 9:**
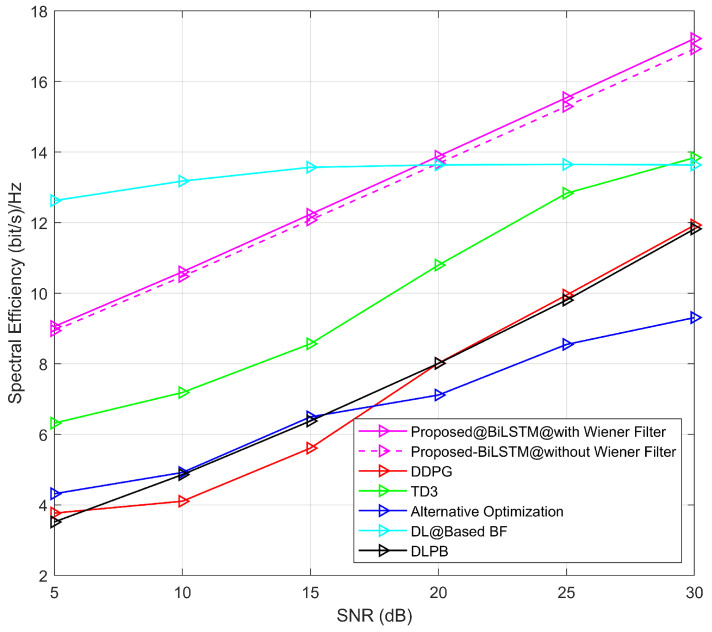
Simulation results of SE for different SNR values are compared with those obtained using DDPG and TD3 [23], Alternative optimization [44], DL@Based BF [24], and DLPB [45].

The conclusive estimated SE result is shown in Figure 9 with different SNR values where the calculated SE is compared with other methods. To test the Wiener filter effectiveness, the simulation result without the filter is presented in Figure 9. From the figure, it is shown that without the filter, the accuracy of SE is degraded with SNR ranges (0–30) dB compared to the result with the filter. In [23], the authors proposed a DDPG and TD3 model to estimate SE. In the case of the DDPG model, SE is less than 4 bps/Hz for 5 dB SNR, and SE is less than 12 bps/Hz for 30 dB SNR.

Similarly, in the case of the TD3 model, SE is 6.4 bps/Hz for 5 dB SNR and almost 14 bps/Hz for 30 dB SNR. The alternative optimization algorithm is applied in [44] to calculate the SE performance. For this technique, SE achieves 4.4 bps/Hz and 9.2 bps/Hz with 5 dB and 30 dB SNR, respectively. The DL-based BF method is also applied to calculate SE in the study [24], where 12.8 bps/Hz SE is calculated for 5 dB SNR and 13.8 bps/Hz SE is calculated for 30 dB SNR. The proposed Bi-LSTM model-based SE performance is also shown in Figure 9. Figure 9 shows that the SE of the proposed model gains 9 bps/Hz and 17.2 bps/Hz for 5 dB and 30 dB SNR, respectively. The graph shows that proposed Bi-LSTM model is better than all other compared models. Only the SE of the DL-based BF model is better than the SE of the proposed model in terms of lower SNR. But in the case of higher SNR, the proposed model SE leads over the DL-based BF model SE. In addition, the proposed method also outperforms the DL-based predictive beamforming (DLPB) [45]. It is concluded that the proposed Bi-LSTM model outperforms DDPG, TD3, DLPB, alternative optimization, and the DL-based BF model.

## 5. Conclusions

In this paper, we proposed an IRS-assisted Bi-LSTM model-based SE calculation in a downlink MU-MISO communication system. The generated dataset was used to train the proposed model in the offline phase, and the learned model was then used in the online phase to test data. As a result, it was possible to extract the transmitted data and determine the CSI parameters. IRS phase shift was optimized with the Wiener filter to determine the optimal phase. Training and validation losses were estimated at 800 epochs and both losses were shown to have very similar trends. After that, we started the model prediction process and compared the prediction accuracy with the ground truth. The model could predict very accurately and with excellent matching of test and predicted values. The performance evaluation of the proposed Bi-LSTM model was obtained by calculating loss with different SNR values. Finally, we estimated the SE for various SNR values and compared them with DDGP, TD3, alternative optimization, and DL-based BF model results. However, the proposed Bi-LSTM network is more reliable than existing methods for SE calculation.The proposed method needs to be tuned with different system parameters and trained with large datasets which can enhance the overall SE of the proposed system. In the future, the proposed model may be a probable solution to the implementation of real-time instruments as well as more progressive systems such as the MIMO communication system. In the deployment phase of the proposed model, it will be easier to implement the proposed Bi-LSTM on the base station, as the power supply in the base station is sufficient and complex hardware can be installed such as GPU easily. However, on the mobile user side, it will be challenging to implement the model as several obstacles need to be addressed. The first is the deep learning model size, which should be reduced before deploying to a mobile device. Also, we need to take into account power consumption to make inferences from the model. These issues are in need of further investigation to ensure optimal performance.

## Figures and Tables

**Table 1:** The notation of Algorithm 2 parameters.

Parameters	Explanation
$fg_{t}$	At iteration *t*, the activation vector of forget gate
$Q_{fg},T_{fg}$	Weight vector for forget gate
$X_{t}$	Input vector of LSTM at iteration *t*
$P_{t-1}$	The hidden state vector at iteration t−1
$b_{fg}$	Bias vector of forget gate
*Q*	Sigmoid activation function
$ig_{t}$	Input gate activation vector at repetition *t*
$Q_{ig},T_{ig}$	Weight vectors of input gate
$b_{ig}$	Bias vector of input gate
$cd_{t}$	Candidate gate activation vector at repetition *t*
$Q_{cd},T_{cd}$	Weight vectors of candidate gate
$b_{cd}$	Candidate gate bias vector
$og_{t}$	Output gate activation vector at repetition *t*
$Q_{og},T_{og}$	Weight vectors of output gate
$b_{og}$	Output gate bias vector
$C_{t}$	The cell state vector at repetition *t*
$P_{t}$	The hidden state vector at repetition *t*
$H_{t}$	The hidden state vector of Bi-LSTM layer output
tanh	Hyperbolic tangent function
${\vec{T}}_{t}$	The Bi-LSTM network forward sequence
${\overset{\leftarrow }{T}}_{t}$	Backward sequence of the Bi-LSTM network

**Table 2:** The simulation parameters of the proposed system.

Parameters	Value
Number of samples	10,000
Number of base antenna	8
Number of transmission antenna	8
Noise type	AWGN
Total model layer	5
Number of LSTM hidden layer	400
Number of IRS elements	8
Learning rate	0.001
Batch size	100
Number of epoch	800
Optimizer	Adam

## Data Availability

Not applicable.
